# A Novel Optimized iBeacon Localization Algorithm Modeling

**DOI:** 10.3390/s23146560

**Published:** 2023-07-20

**Authors:** Zhengyu Yu, Liu Chu, Jiajia Shi

**Affiliations:** 1 School of Transportation and Civil Engineering, Nantong University, Nantong 226019, China; zheng.y.yu@hotmail.com (Z.Y.); julie.chu.cl@gmail.com (L.C.); 2 Faculty of Engineering and Information Technology, University of Technology Sydney, Ultimo, NSW 2007, Australia

**Keywords:** iBeacon localization, Kalman filter, Bluetooth low energy, wireless sensor networks, received signal strength indicator

## Abstract

The conventional methods for indoor localization rely on technologies such as RADAR, ultrasonic, laser range localization, beacon technology, and others. Developers in the industry have started utilizing these localization techniques in iBeacon systems that use Bluetooth sensors to measure the object’s location. The iBeacon-based system is appealing due to its low cost, ease of setup, signaling, and maintenance; however, with current technology, it is challenging to achieve high accuracy in indoor object localization or tracking. Furthermore, iBeacons’ accuracy is unsatisfactory, and they are vulnerable to other radio signal interference and environmental noise. In order to address those challenges, our study focuses on the development of error modeling algorithms for signal calibration, uncertainty reduction, and interfered noise elimination. The new error modeling is developed on the Curve Fitted Kalman Filter (CFKF) algorithms. The reliability, accuracy, and feasibility of the CFKF algorithms are tested in the experiments. The results significantly show the improvement of the accuracy and precision with this novel approach for iBeacon localization.

## 1. Introduction

Indoor localization technology is a crucial area of study within the realm of Wireless Sensor Networks (WSN) [1,2,3]. Current research widely uses the Received Signal Strength Indicator (RSSI) in WSN because of its ease of operation; however, in indoor environments, RSSI-based location measurement is impacted by multipath fading effects [4]. Signal transmission is interfered with by furniture, people, and objects; this results in poor accuracy [5,6,7]. Sensor node localization or range-based localization techniques calculate the distance between the nearest nodes [8,9]; they are used in most WSN applications [10]. The node localization method in WSN typically measures range using the RSSI [11,12]. Previous studies [13] have explored various technologies for indoor localization, including RFID [14,15], RADAR [16], WiFi [17], and 5G [18]; however, these technologies need to offer the essential balance of low cost, low energy consumption, accuracy, reliability, and ease of use. Bluetooth is a low-cost wireless sensor for short-distance communication [19,20]. Bluetooth Low Energy (BLE) is a crucial technology that enables the use of iBeacon [7,20,21]. BLE-mounted beacons contain identification information to detect the location of the system [22]. iBeacon is a power-efficient and cost-effective indoor localization system that can send presence information to iOS 7 or above devices and Android systems [23,24,25]. The indoor localization system uses identification from the sensor device and the data of the RSSI from a smartphone [26]. The range between the iBeacon and the smartphone is determined by the value of the estimated RSS [27]. The RSS values are highly susceptible to errors caused by factors such as the transmission platform, the surrounding environment, and other communication devices such as receivers and antennas [22,28]. The RSSI technique is primarily used to obtain the signal strength from Bluetooth and iBeacon and identify the localization data [29]. A localization method based on Bayes’ likelihood algorithm with RSSI and fingerprint is proposed to approach the accuracy at meter level in a static environment. The accuracy can be improved by 20% but the fingerprint training process takes much longer and is difficult [30]. A machine learning algorithm applying the RSSI-based fingerprinting method was introduced recently. The offline performance is well-presented whereas the online performance is not comprehensive due to a lack of calibration [31]. The least squares methods, such as nonlinear least squares, total least squares, weighted least squares, and generalized least squares, etc., are utilized to analyze the transmission model for Bluetooth localization accuracy. The results indicate that the generalized least squares method performs with better accuracy than the others [32]. The KF is a recursive linear filtering technique that can be utilized to minimize the impact of noise in position measurements [33]. It processes past measurements to constrain arbitrary calculation deviations [34]. A Bluetooth signal strength measurement method using an Extended Kalman Filter (EKF) is proposed to obtain the distance accuracy [35]. The weighted least square algorithm is used in the KF equations to reduce the square error for the variance. The developed KF can smooth the noise caused by signal drift and vibration [36]; however, the calibration process is not introduced in the research. The developed KF method does not apply to the measured distance compared with the estimated distance, which could achieve better distance accuracy. A solution that meets all these requirements has yet to be discovered. Improving the accuracy and precision of localization in iBeacon can be achieved by implementing error modeling algorithms based on a developed KF with a least squares algorithm to correct distance measurement errors.

This paper introduces novel error modeling algorithms for indoor localization. Based on a CFKF error-modeling filter-adjustment strategy, the proposed methodology reduces measurement errors in the iBeacon localization system. The CFKF error modeling algorithm considers iBeacon sensor calibration, improving the measured data through error modeling. This research compares the proposed CFKF error modeling with recent existing error filtering methods according to accuracy and precision. The experimental results show that the CFKF error modeling approach provides the most accurate and precise results for iBeacon localization. The main contributions of the article are:

- A developed KF state estimate algorithm based on the modified Least Squares Algorithm (LSA) is proposed to enhance the accuracy of iBeacon measurement distance;
- A developed system calibration process has been proposed to calibrate the RSSI and estimated distance for indoor localization using iBeacon;
- A novel CFKF error modeling approach has been proposed to enhance the estimation accuracy of iBeacon systems in field experiments.

The paper is structured as follows: Section 2 introduces the materials and methods for the research, including the iBeacon protocol, calibration process, and CFKF algorithms. Section 3 states the results and discussions of the iBeacon localization experiment, including the system calibration, error modeling calculations, and field experiment. Finally, conclusions are presented in Section 4.

## 2. Materials and Methods

### 2.1. iBeacon Protocol

The iBeacon system utilizes a localization technique with the BLE method to determine the objects’ locations [37]. The distance between an object and iBeacon is calculated by detecting when the object enters or exits a designated position and measuring the Bluetooth’s signal transmission and Received Signal Strength (RSS). The information transmitted by iBeacon includes a Universally Unique Identifier (UUID) that distinguishes each company. Major information represents a group of related iBeacons, and Minor information represents the specific iBeacon. The code area in iBeacon, which is 4 bytes, can only be used to identify a specific area within a building, not individual objects [38].

#### 2.1.1. UUID

The iBeacon employs an identifier in 128-bit known as UUID for transmitting its information. The UUID holds information about the organization that the iBeacon belongs to, thus identifying the company that it is associated with [39]. In the Bluetooth specification, UUID is used as a unique identifier for some devices. The UUID is situated at the highest level of the hierarchy and serves as an identifier for the ordinary management of iBeacons. Unlike some networking protocols, such as 802.11, Bluetooth does not have central management of UUIDs to prevent conflicts. Instead, iBeacons can generate a random unique UUID at any moment [39].

#### 2.1.2. BLE Minor Number

The Minor number is employed to distinguish a lower-level group within a set of iBeacons. As an illustration, in a retail chain, it can be utilized to recognize a specific item within a single shop [40].

#### 2.1.3. BLE Major Number

The Major number, as specified in Bluetooth and iBeacon specifications, is utilized to identify the ID of a particular class of iBeacons that belong to a particular body. As an illustration, in a retail chain, it is utilized to distinguish the particular shop in which a particular class of beacons is situated [39].

#### 2.1.4. Advertising Interval

The advertising interval is typically set to 100 milliseconds in Bluetooth. While extending the advertising interval can increase battery life, it also decreases the frequency of signal transmission in communication, requiring a balance to be struck between the length of the advertising interval and the intended usage [41].

### 2.2. Calibration Process

The range measurement accuracy in iBeacon depends on the calibration constant, “measured single power”. Smartphones use the constant to determine the RSS. It is assumed that the iBeacon is located in free space, with signal loss primarily impacted by inverse-square fading; however, in real-world experimentation, the measured range experiences fluctuations due to interference caused by multiple paths. When the RF signal is transmitted through multiple pathways, the radio energy can interfere with other radio transmissions. This interference can either strengthen the signal (constructive interference) or weaken it (destructive interference). Multipath interference often occurs when the iBeacon is placed near the ceiling or a barrier, causing the radio signal to reflect when it hits the surface.

**Figure 1:**
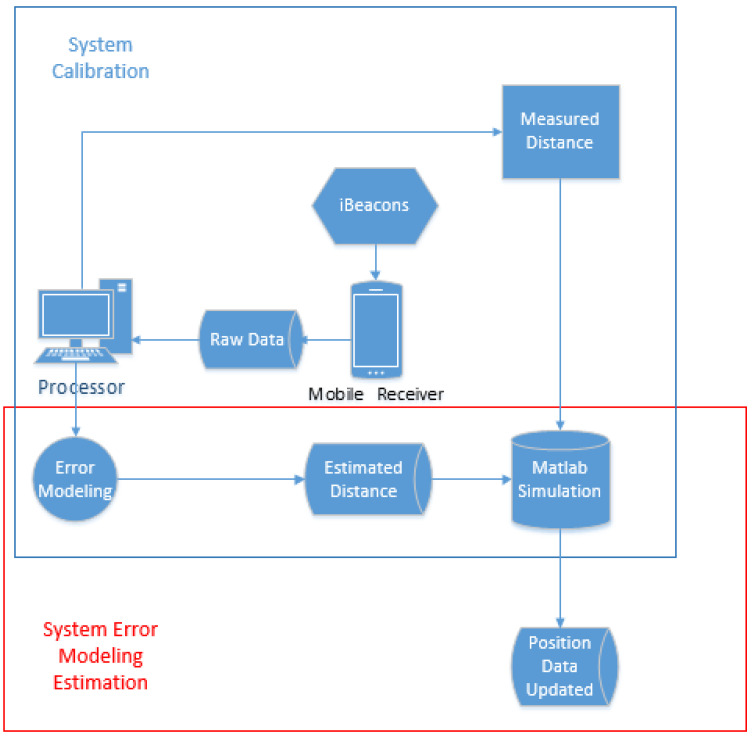
System Calibration and Error Modeling Estimation.

As shown in Figure 1, the system calibration stage focuses on initializing RSSI at a 1 m distance. The experiment involves the iBeacon broadcasting raw RSSI data signals while a smartphone with Bluetooth is the mobile receiver. The data from the iBeacon is received by the Bluetooth of the mobile receiver, and the raw RSSI data is recorded and stored on the mobile receiver. The stored data, which includes time (milliseconds), UUID, Minor number, Major number, and RSSI (dBm), is then transferred to a computer for analysis. Equation (1) is used to process the measured power at a 1 m distance from the iBeacon.

(1) $$A=RSSI+\left[N\cdot 10\;\mathrm{lg}\left(D\right)\right]$$

where D denotes distance;

RSSI denotes signal strength, RSSI;
A denotes measured power at a 1 m distance;
N denotes environmental factor, N = 2 in free space.

We need to know the values of RSSI and A to determine the values of distance. The RSSI value can be collected from the raw data logged by the mobile receiver. The value of the constant “A” can be determined by positioning the iBeacon 1 m away from the mobile receiver. This calibration procedure helps to minimize noise and errors in the signal transfer. After collecting the raw data, the data must be processed using error modeling algorithms, such as KF, Curve Fitting (CF), or CFKF, to generate an estimated value. The environmental factor, N, represents the signal loss due to distance and environment. In free space, N is equal to 2, and, generally, it does not change as long as the environment remains the same; however, in case of a change in the environment, N can be calculated using Equation (2).

(2) $$N=\left(A-RSSI\right)/\left(10\;\mathrm{lg}\left(D\right)\right)$$

The next step after obtaining the estimated data is to plot it on a Matlab figure. The system then calculates the error rates, revealing that the CFKF algorithm is the most accurate and precise among the others. This completes the calibration stage. To calculate the distance between the iBeacon and the mobile receiver at a value other than 1 m using the received RSSI, the previous results, including the value of A, must be utilized in Equation (3).

(3) $$D=10^{\left(\frac{A-RSSI}{10N}\right)}$$

To calculate a distance different from 1 m using the RSSI value, the previous results, including the value of A, must be utilized in Equation (3). The accuracy of the different algorithms in calculating distance at various ranges is evaluated by plotting the results in Matlab.

**Figure 2:**
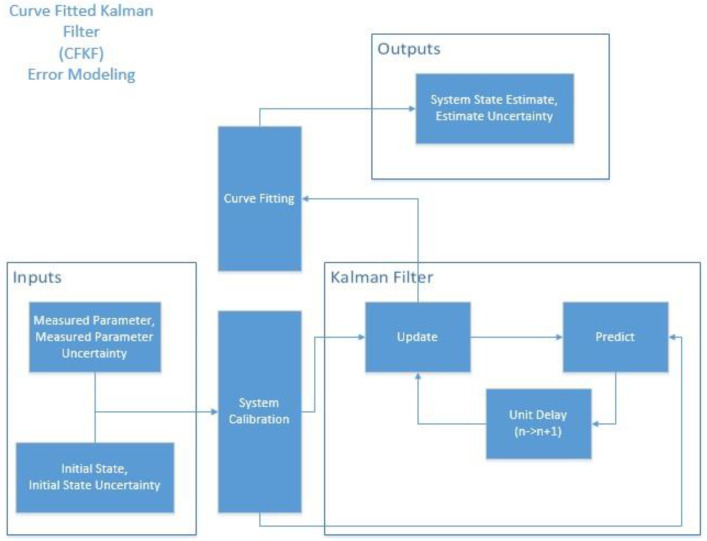
CFKF Error Modeling Workflow Diagram.

In Figure 2, the inputs used for calculation are the measured distances obtained from Equation (3) based on raw data. These inputs are programmed using the KF. The updated data generated by KF is not the most accurate and will be further calculated using Curve Fitting (CF) to produce the final estimated data, referred to as the CFKF-estimated data. The equations for CFKF modeling are as follows:

(4) $$y_{i}=\beta_{0}+\beta_{1}x_{i}+\beta_{2}{x_{i}}^{2}+\cdots +\beta_{k}{x_{i}}^{k}+\varepsilon_{i}\left(i=1,2,\ldots ,n\right)$$

(5) $$y=\left[\begin{array}{c} y_{1}\\ y_{2}\\ \begin{array}{c} y_{3}\\ \begin{array}{c} \vdots \\ y_{n} \end{array} \end{array} \end{array}\right]$$

(6) $$X=\left[\begin{array}{cc} \begin{array}{c} 1\\ 1\\ 1 \end{array} & \begin{array}{ccc} x_{1} & x_{1}^{2} & \begin{array}{cc} \vdots & x_{1}^{k} \end{array}\\ x_{2} & x_{2}^{2} & \begin{array}{cc} \vdots & x_{2}^{k} \end{array}\\ x_{3} & x_{3}^{2} & \begin{array}{cc} \vdots & x_{3}^{k} \end{array} \end{array}\\ \begin{array}{c} \vdots \\ 1 \end{array} & \begin{array}{c} \begin{array}{ccc} \begin{array}{cc} \vdots & \vdots \end{array} & \ddots & \vdots \end{array}\\ \begin{array}{ccc} x_{n} & x_{n}^{2} & \begin{array}{cc} \vdots & x_{n}^{k} \end{array} \end{array} \end{array} \end{array}\right]$$

(7) $$\beta =\left[\begin{array}{c} \beta_{0}\\ \beta_{1}\\ \begin{array}{c} \beta_{2}\\ \begin{array}{c} \vdots \\ \beta_{k} \end{array} \end{array} \end{array}\right]$$

(8) $$\;\varepsilon =\left[\begin{array}{c} \varepsilon_{1}\\ \varepsilon_{2}\\ \begin{array}{c} \varepsilon_{3}\\ \begin{array}{c} \vdots \\ \varepsilon_{n} \end{array}\; \end{array} \end{array}\right]$$

(9) y=Xβ+ε

For optimal compatibility with the Least Square Algorithm (LSA), the algorithm is initialized with k=1, $\beta_{0}=0$ as follows:

(10) $$y_{i}=\beta x_{i}+\varepsilon \;$$

where β denotes the scale factor of the system;

ε denotes the bias of the system.

Here, the KF State Update Estimate equation is modified by incorporating the Least Square Algorithm (LSA):

(11) $$\begin{array}{cc} {\hat{y}}_{n,n} & =\beta {\hat{x}}_{n,n}+\varepsilon ={\beta \hat{x}}_{n,n-1}+{\beta K}_{n}\left(z_{n}-{\hat{x}}_{n,n-1}H\right)+\varepsilon \\ & =\beta \left(1-K_{n}H\right){\hat{x}}_{n,n-1}+{\beta K}_{n}z_{n}+\varepsilon \end{array}$$

At this stage, the distance measurement values are calculated using Equation (3) after processing and calibrating the raw data collected by the mobile receiver using the CF algorithm to determine A. The state of the object is then updated using a state update equation, which is as follows:

(12) $${\hat{x}}_{n,n}={K_{n}\left(z_{n}-{\hat{x}}_{n,n-1}\right)+\hat{x}}_{n,n-1}=K_{n}z_{n}+(1-K_{n}){\hat{x}}_{n,n-1}$$

(13) $$z_{n}=D=10^{\left(\frac{A-RSSI}{10N}\right)}$$

(14) $${\hat{x}}_{n,n}=K_{n}\cdot 10^{\left(\frac{A-RSSI}{10N}\right)}+(1-K_{n}){\hat{x}}_{n,n-1}$$

According to the derivation of LSA:

(15) $$\;\beta =\frac{\sum \left({\hat{x}}_{n,n}{\hat{y}}_{n,n}\right)-\sum \left({\hat{x}}_{n,n}\right)\sum \left({\hat{y}}_{n,n}\right)/n}{\sum \left({{\hat{x}}_{n,n}}^{2}\right)-{\left(\sum {\hat{x}}_{n,n}\right)}^{2}/n}$$

(16) $$\varepsilon =\frac{\left(\sum {{\hat{x}}_{n,n}}^{2}\right)\left({\sum \hat{y}}_{n,n}\right)-\left(\sum {\hat{x}}_{n,n}\right)\left(\sum {{\hat{x}}_{n,n}\hat{y}}_{n,n}\right)}{n\left(\sum {{\hat{x}}_{n,n}}^{2}\right)-{\left(\sum {\hat{x}}_{n,n}\right)}^{2}}$$

Therefore,

(17) $${\hat{y}}_{n,n}=\frac{\sum \left({\hat{x}}_{n,n}{\hat{y}}_{n,n}\right)-\sum \left({\hat{x}}_{n,n}\right)\sum \left({\hat{y}}_{n,n}\right)/n}{\sum \left({{\hat{x}}_{n,n}}^{2}\right)-{\left(\sum {\hat{x}}_{n,n}\right)}^{2}/n}{\hat{x}}_{n,n}+\frac{\left(\sum {{\hat{x}}_{n,n}}^{2}\right)\left({\sum \hat{y}}_{n,n}\right)-\left(\sum {\hat{x}}_{n,n}\right)\left(\sum {{\hat{x}}_{n,n}\hat{y}}_{n,n}\right)}{n\left(\sum {{\hat{x}}_{n,n}}^{2}\right)-{\left(\sum {\hat{x}}_{n,n}\right)}^{2}}$$

The new State Update equation for KF is formulated as follows:

(18) $${\hat{y}}_{n,n}=\frac{\sum \left({\hat{x}}_{n,n}{\hat{y}}_{n,n}\right)-\sum \left({\hat{x}}_{n,n}\right)\sum \left({\hat{y}}_{n,n}\right)/n}{\sum \left({{\hat{x}}_{n,n}}^{2}\right)-{\left(\sum {\hat{x}}_{n,n}\right)}^{2}/n}\left(1-K_{n}H\right){\hat{x}}_{n,n-1}+\frac{\sum \left({\hat{x}}_{n,n}{\hat{y}}_{n,n}\right)-\sum \left({\hat{x}}_{n,n}\right)\sum \left({\hat{y}}_{n,n}\right)/n}{\sum \left({{\hat{x}}_{n,n}}^{2}\right)-{\left(\sum {\hat{x}}_{n,n}\right)}^{2}/n}K_{n}\cdot 10^{\left(\frac{A-RSSI}{10N}\right)}\;+\frac{\left(\sum {{\hat{x}}_{n,n}}^{2}\right)\left({\sum \hat{y}}_{n,n}\right)-\left(\sum {\hat{x}}_{n,n}\right)\left(\sum {{\hat{x}}_{n,n}\hat{y}}_{n,n}\right)}{n\left(\sum {{\hat{x}}_{n,n}}^{2}\right)-{\left(\sum {\hat{x}}_{n,n}\right)}^{2}}$$

The Kalman Gain equation:

(19) $$K_{n}=\frac{P_{n,n-1}H^{T}}{HP_{n,n-1}H^{T}+R_{n}}$$

A covariance update equation is presented below:

(20) $$P_{n,n}=K_{n}R_{n}K_{n}^{T}+\left(I-K_{n}H\right)P_{n,n-1}{\left(I-K_{n}H\right)}^{T}$$

## 3. Result and Discussions

### 3.1. iBeacon Localization Experiment

The experiment for the iBeacon localization system has been conducted in this section. The initial step was to calibrate the system, and multiple algorithms were evaluated to determine the most effective one for maximizing localization accuracy. The measurement phase was then carried out to determine the estimated distance. Finally, an experiment was conducted in a real-life environment. The results indicated that the error modeling using CFKF provided the highest accuracy for the system.

#### 3.1.1. iBeacon Localization Testbed Setup

The experiment utilized Estimote iBeacons, and their specifications can be found in Table 1.

The specifications of the smartphone can be found in Table 2.

#### 3.1.2. Error Modeling Calibration Process for Distance Measurement

**Figure 3:**
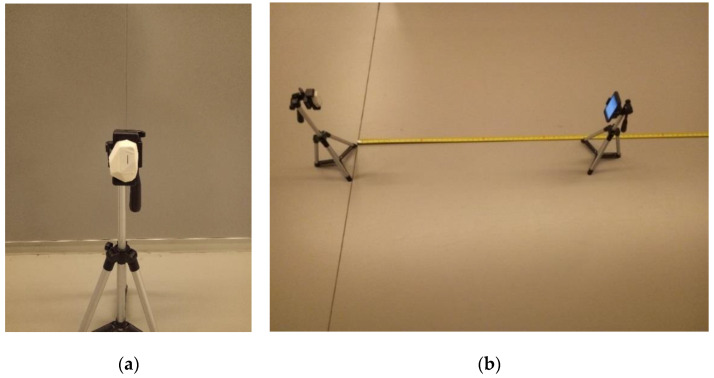
Error Modeling Calibration Testbed (**a**,**b**).

As shown in Figure 3a,b, both the iBeacon and mobile receiver were fixed on stands that stood 50 cm tall and positioned 1 m apart. This test setup was used to calibrate the distance measurement for the iBeacon.

The datasets received by the mobile phone from the iBeacon can be found in the repository [42]. The datasets include the time (milliseconds), UUID, Minor number, Major number, and RSSI (dBm). The signal transmission interval was around 300 milliseconds, and the device was placed at a distance of 1 m from the mobile phone receiver to gather data. A total of 1500 datasets were gathered; below are a few examples.

**Figure 4:**
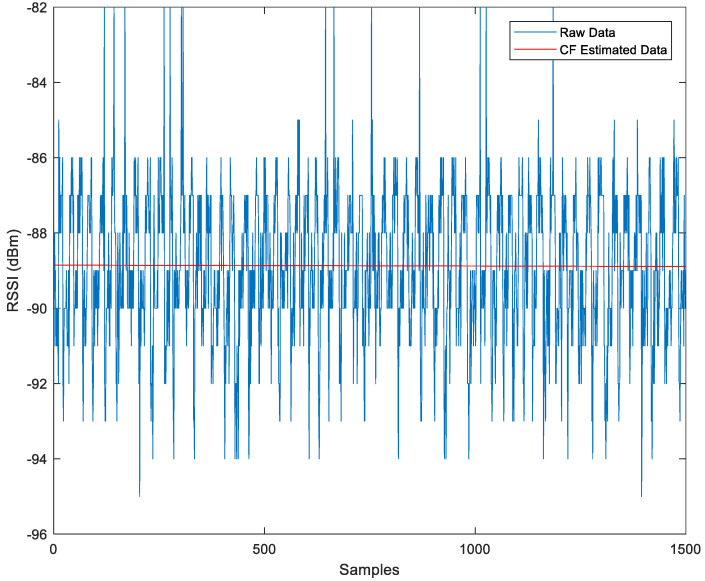
Comparison Between Raw Data and CF-Estimated Data.

Figure 4 illustrates the linear modeling of 1500 sample sets of RSSI (${x_{r}}_{n,n}$) using the CF algorithm. The blue lines depict the raw RSSI data (${y_{r}}_{n,n}$), and the red line shows the CF data. The $\beta_{r}$ in the modeling represents the scaling of the CF data, while $\varepsilon_{r}$ represents the measured power at 1 m. The CF algorithm is also displayed.

CF error modeling for raw data (CF-RSSI):

(21) $${y_{r}}_{n,n}\;=\;\beta_{r}\;{x_{r}}_{n,n}\;+\;\varepsilon_{r}$$

$$\;\;\beta_{r}=-\mathrm{2.23}\times {\mathrm{10}}^{-5}\;(-\mathrm{0.000246},\mathrm{0.000199})$$

($\beta_{r}$ is −2.23 × 10^−5^, the mean of the range between −0.000246 and 0.000198)

$$\varepsilon_{r}=-\mathrm{88.85}(-\mathrm{89.04},-\mathrm{88.67})$$

When the value of $\varepsilon_{r}$ is −88.85, the coefficient $\beta_{r}$ is −2.23 × 10^−5^, which is too tiny to affect the results; therefore, the influence of $\beta_{r}\;$can be omitted, whereas the value of $\varepsilon_{r}$ accounts for much more.

(ε is −88.85, the mean of the range between −89.04 and −88.67)

**Figure 5:**
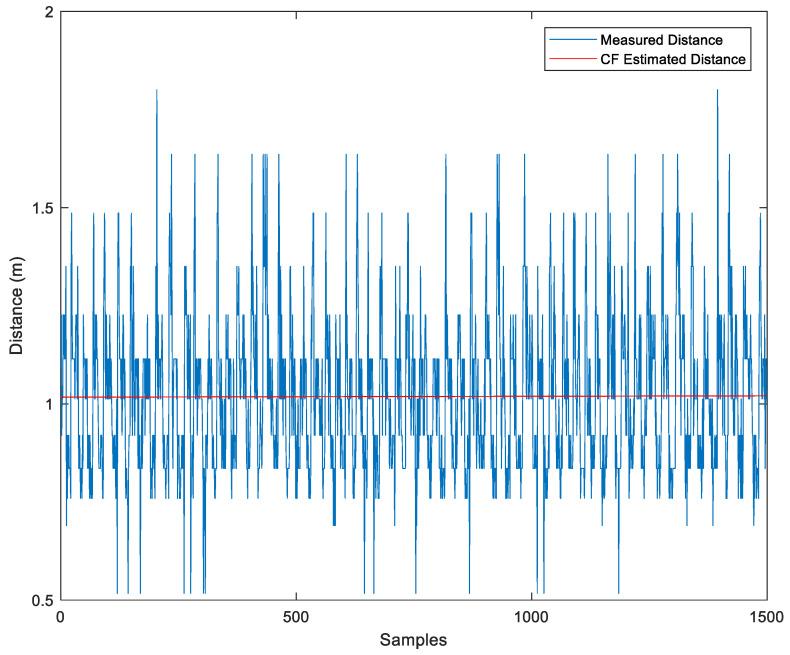
Comparison Between Measured Distance and CF-Estimated Distance.

Figure 5 displays the conversion of the raw data’s RSSI into distance using Equation (3), $D=10^{\left(\frac{A-RSSI}{10N}\right)}$, with A set to the value of $\varepsilon_{r}$ = −88.85, the calibrated value of N is 2.4. The raw data’s distance values and the distance values estimated by the CF algorithm are shown.

Where $\beta_{d}$ represents the scale factor and $\varepsilon_{d}$ represents the bias of the estimated distance calculated by the CF algorithm, the error modeling for the CF-estimated distance can be expressed as follows:

(22) $${y_{d}}_{n,n}\;=\beta_{d}\;{x_{d}}_{n,n}\;+\varepsilon_{d}$$

$$\beta_{d}=\mathrm{0.00121}(-\mathrm{0.0108},\mathrm{0.0133})$$

$$\varepsilon_{d}=\mathrm{1.027}(\mathrm{1.015},\mathrm{1.039})$$

**Figure 6:**
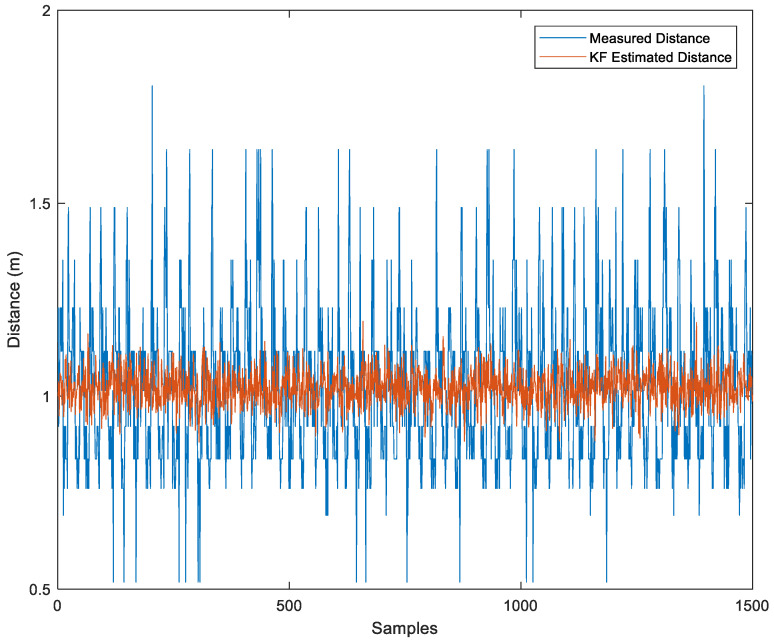
Comparison Between Measured Distance and KF-Estimated Distance.

Alternatively, we can use the KF to reduce noise in the measured distance. As shown in Figure 6, the KF estimated value is displayed in red.

**Figure 7:**
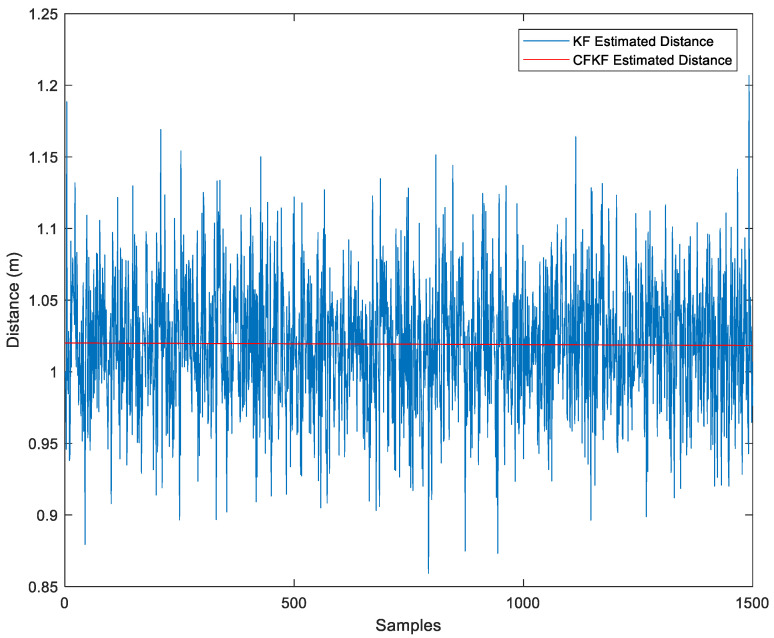
Comparison of Estimated Distance using KF and CFKF.

Finally, the CF algorithm was applied to the KF-estimated distance, resulting in even greater accuracy, as depicted in Figure 7.

The CFKF error modeling for the distance (CFKF-Distance) is computed using Equation (11),$\;{\hat{y}}_{n,n}$ *=* β ${\hat{x}}_{n,n}$ *+* ε, and the results are displayed as follows:

β=−0.00154 (−0.00402,0.000953)

ε=1.026 (1.023,1.028)

#### 3.1.3. Error Modeling Optimized Calibration Results

**Figure 8:**
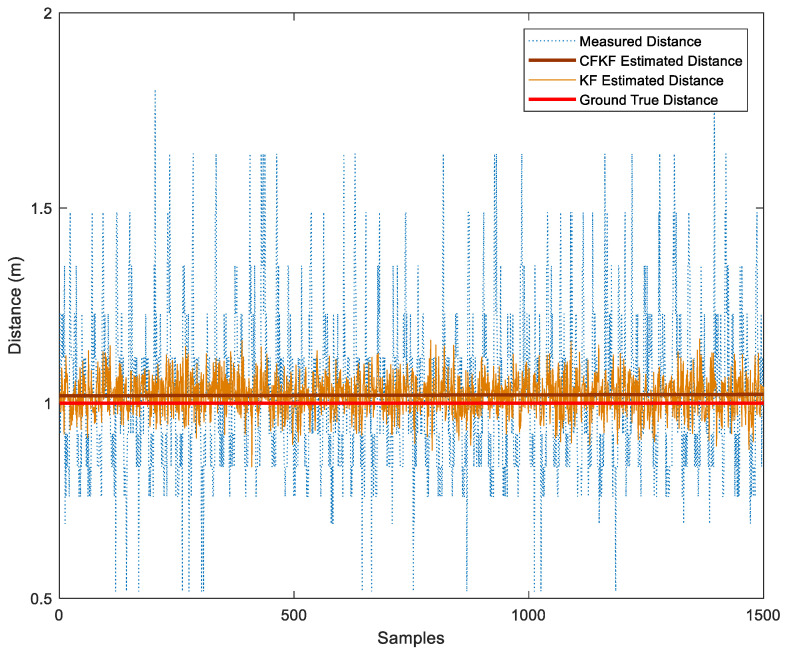
Comparison Results of Error Modeling Calibration.

The algorithm bias and Mean Absolute Error (MAE) in Table 3 show that the CFKF-estimated distance is more accurate and precise than other algorithms. The results can be seen in Figure 8, which show that the distance estimated through the CFKF method successfully minimizes uncertainties and is much closer to the actual distance than other methods.

**Figure 9:**
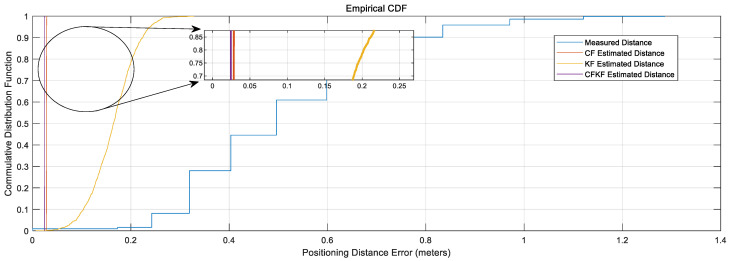
CDF Comparison Results for Calibration.

From Figure 9, the comparison CDF plots present that our CFKF estimated result outperforms the other algorithms.

**Figure 10:**
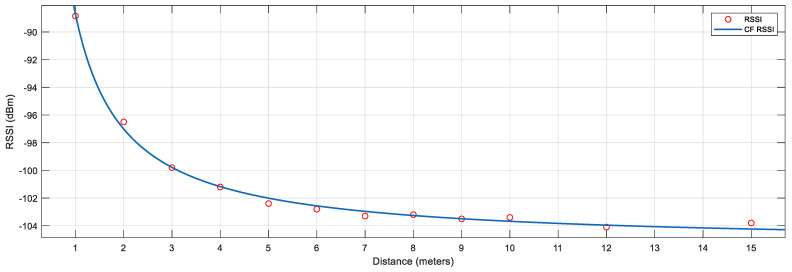
RSSI vs. Distance from 1 to 15 m.

We repeated the calibration processes from 1 m to 15 m. Same as the previous calibration, 1500 raw data samples were collected and processed for each distance. The calibrated RSSI values have been recorded in Table 4. Figure 10 presents the RSSI values and curve-fitted RSSI values. When the distance is larger than 8 m, the difference in the RSSI values is minor due to the loss of signal strength. The signal strength is poor when RSSI is less than −103.5 dbm. Due to the limitation of the device, the distance between the iBeacon and the receiver should be no more than 8 m to obtain the best accuracy. We can also extend the iBeacon setup for every 8 m to measure the longer distance.

#### 3.1.4. Field Experiment of iBeacon Localization

**Figure 11:**
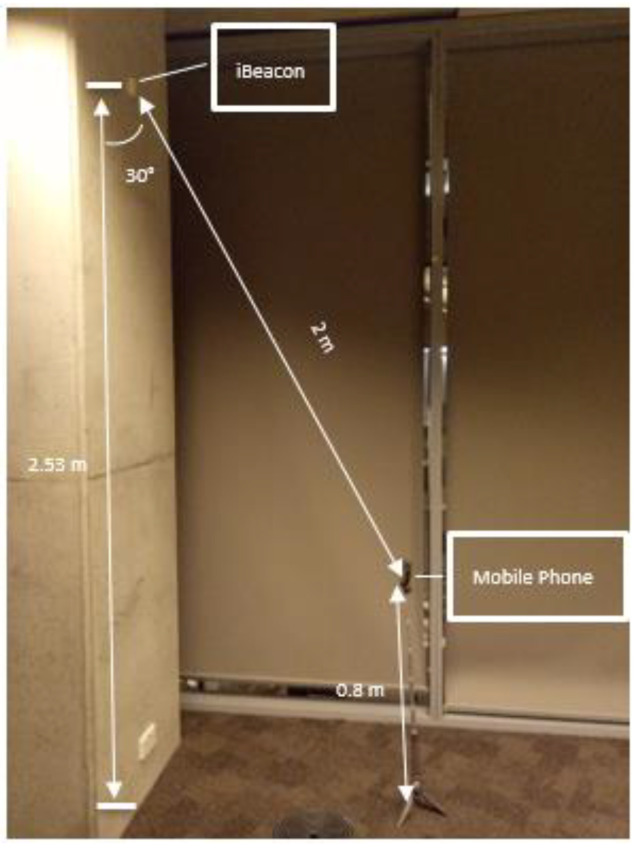
Testbed for iBeacon Localization Field Experiment.

As illustrated in Figure 11, the iBeacon was placed 2.53 m above the wall of an office. To avoid signal reflection interfering with signals transmitted from other angles, the iBeacon was positioned 0.2 m away from the ceiling. A setup was created, which consisted of a stand with a mobile phone on top, positioned 1 m away from the wall and 0.8 m in height. As shown in Figure 11, it was estimated that the angle between the wall and the line connecting the iBeacon and the mobile phone was roughly 30 degrees, and the distance between the two devices was approximately 2 m. This was a real-world field experiment, and the CFKF error modeling process results were validated through calculation.

**Figure 12:**
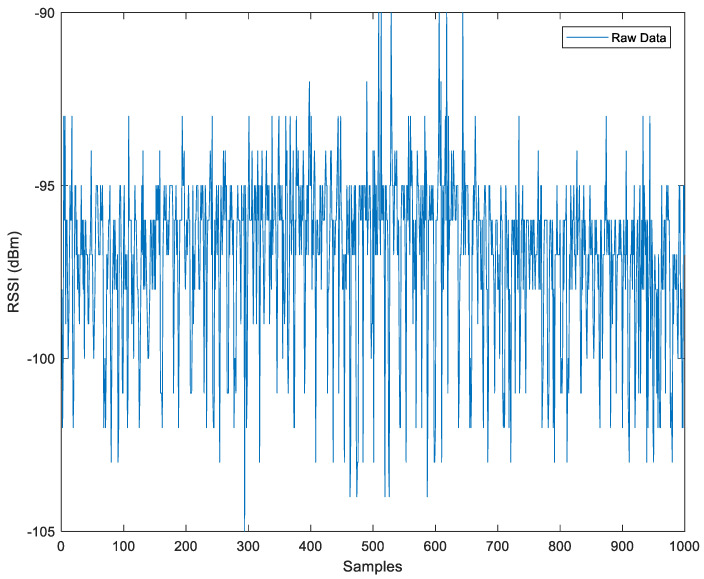
RSSI Raw Data for the Field Experiment.

Figure 12 displays 1000 samples of RSSI received by the mobile phone. Using Equation (20), we can calculate the measured power from the iBeacon at an angle of 30° (or 150° from the top).

In this scenario, the measured distance was determined using Equation (3), and the CF and CFKF error modeling is processed using Equations (11) and (21), respectively. The results are presented as follows:

CF error modeling for the distance (CF-Distance)

$$\beta_{d}=0.0671\;(0.0371,0.0971)$$

$$\varepsilon_{d}=2.098\;(2.057,2.139)$$

CFKF error modeling for the distance (CFKF-Distance):

$$\beta =-3.35\times 10^{-6}\;(-8.74\times 10^{-6},2.03\times 10^{-6})$$

ε=2.09 (2.084,2.095)

The comparison of the results between the KF algorithm and the CFKF algorithm can be seen in Figure 11, shown in the distance diagram.

**Figure 13:**
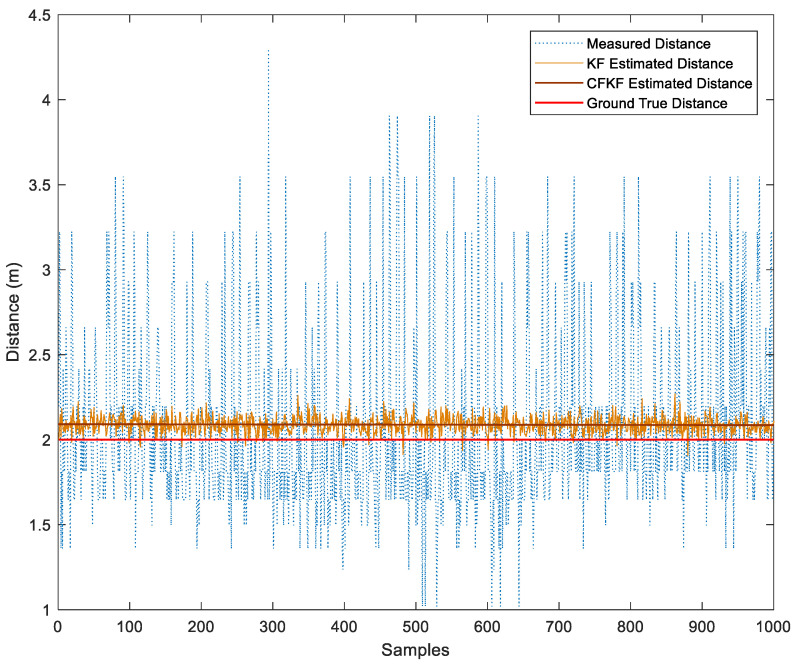
Distance Comparison Results of Error Modeling for Field Experiment.

**Figure 14:**
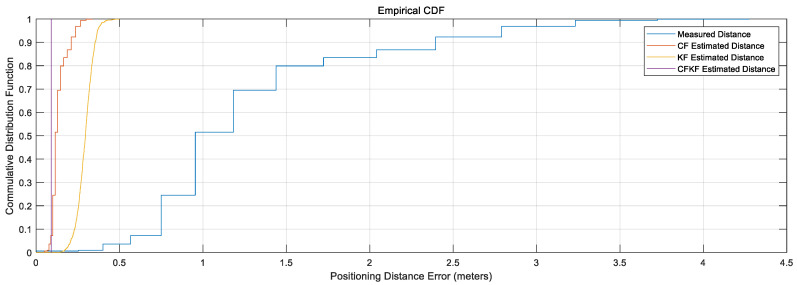
CDF Comparison Results for Field Experiment.

As demonstrated in Figure 13, the CFKF-estimated distance is the significantly closest result to the actual distance of 2.09 m. The KF-estimated distance result is from 1.89 to 2.31 m. The measured distance ranges from 1.019 to 3.55 m. Figure 14 displays that the CFKF-estimated distance is the most accurate in the CDF comparison results, which demonstrates the effectiveness and reliability of our error modeling in the real-life field experiment.

According to Table 5, we can see the MAE of CFKF is less than any others. The bias of the measurement distances varies from 0.35 to 0.67 m. On the other hand, the KF-estimated bias falls in the range of 0.06 to 0.15 m. The CF bias stands at 0.049 m. Meanwhile, the bias of the CFKF is 0.045 m. The results show that the CFKF error modeling provides higher accuracy and precision than the other methods. The CFKF error modeling was discovered to be approximately 30–60% more accurate than the measured results, outperforming the KF estimated distance by 1–10% and surpassing the CF-estimated distance by 0.4%. It shows that the CFKF error modeling provides the highest degree of accuracy.

#### 3.1.5. Large Area Field Experiment of iBeacon Localization

**Figure 15:**
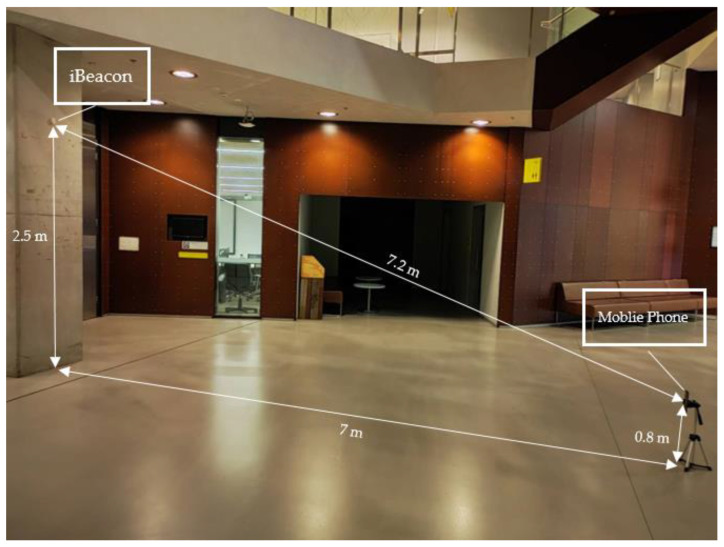
Testbed for iBeacon Localization Field Experiment in the Large Area.

At this stage, we set up the experiment to validate the feasibility of our error modeling in an ample area, as illustrated in Figure 15. The iBeacon was placed 2.5 m above the wall in a large open space. A test bed was set up, which consisted of a stand with a mobile phone on top, placed 7 m away from the wall and 0.8 m in height. From Figure 15, we can estimate that the distance between the iBeacon and the mobile phone was approximately 7.2 m. This was a large-area field experiment for indoor localization.

**Figure 16:**
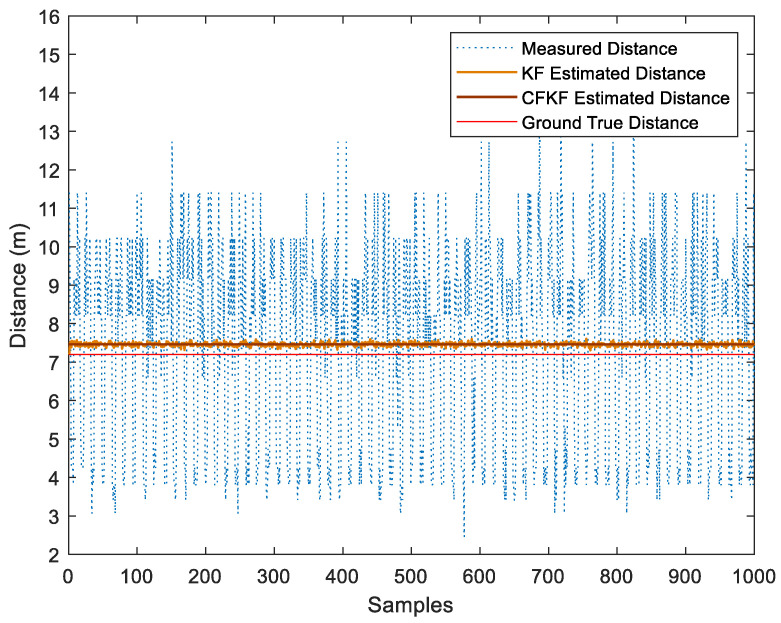
Comparison Results of Error Modeling for Field Experiment in the Large Area.

In this case, we repeated the calibration and calculation process with 1000 received data sets. The comparison of the results among the multiple algorithms is shown in the distance diagram in Figure 16.

**Figure 17:**
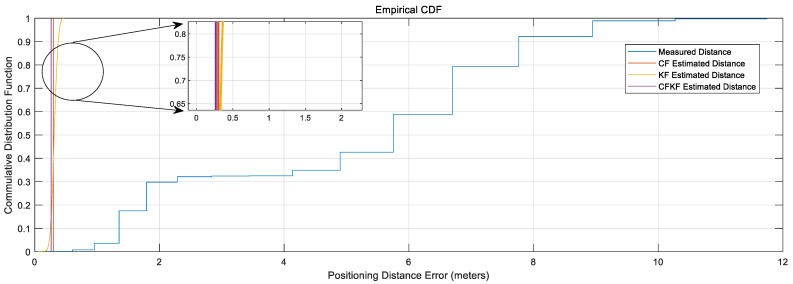
CDF Comparison Results for Long Distance Field Experiment in the Large Area.

As presented in Figure 16, the CFKF-estimated distance is 7.46 m which is the best outstanding result compared to the others. The KF-estimated distance result is from 7.31 to 7.63 m; it needs to be more precise. The measured distance ranges from 2.5 to 12.9 m; it is far from accurate. Moreover, the result from CF was the second-closest result from the ground true value. Figure 17 displays that the CFKF-estimated distance is the most accurate in the CDF comparison results, which demonstrates the effectiveness and reliability of our error modeling in the large-area field experiment.

According to Table 6, the MAE of CFKF is 0.26 m, which is less than any other algorithm. The bias of the measurement distances changes from 4.72 to 5.71 m. The bias of KF starts in the range of 0.11 to 0.45 m; the average value is 0.27 m. The CF bias stands at 0.28 m. Furthermore, the bias of the CFKF is 0.26 m. These results indicate that the CFKF error modeling provides the best accuracy and precision in the large area field experiment.

## 4. Conclusions

In this study, the development and experimentation of CFKF error modeling in iBeacon localization research have been carried out. Firstly, the experimental setup was established, and the iBeacon was positioned a meter away from the mobile receiver during the distance measurement error modeling calibration. The first step was to utilize the CF to process the raw data and calculate the measured power at 1 m. Subsequently, the measured distance was calculated by the distance equation using the received RSSI. To verify the accuracy, various algorithms were tested in the field experiments, with the results indicating that the CFKF error modeling provided the best precision and accuracy among all of them. This CFKF approach can be utilized in RSSI-based iBeacon/Bluetooth localization. In future research, machine learning algorithms could be combined with CFKF error modeling to improve localization performance. The state-of-the-art literature introduces machine learning algorithms based on RSS fingerprinting methods [31,43]. Our error modeling can enhance the calibration process of the online system, which will be demonstrated to improve the result to be more solid and robust. Data fusion techniques are another excellent approach for the future; recent research [44] proposes a method for the fusion of RSSI and magnetometer measurements. The CFKF will be adapted into more sensor networks, such as WIFI, RADAR, and laser range systems, to optimize the localization system’s accuracy.

## Figures and Tables

**Table 1:** iBeacon Specifications.

ARM^®^ Cortex^®^-M4 32-bit processor with FPU
Core speed: 64 MHz
Flash memory: 512 kB
RAM: 64 kB
Radio: 2.4 GHz transceiver
Bluetooth^®^: 4.2 LE standard
Range: up to 200 m (650 feet)
Output Power: −20 to +4 dBm
Frequency range: 2400 to 2483.5 MHz
Modulation: GFSK (FHSS)
Antenna: PCB Meander, Monopole
Over-the-air data rate: 1 Mbps
Length: 62.7 mm
Width: 41.2 mm
Height: 23.6 mm
Weight: 67 g

**Table 2:** Smart Phone Wireless Sensors’ Specifications.

WLAN	Wi-Fi 802.11 a/b/g/n, dual-band, hotspot
Bluetooth	V4.0
GPS	A-GPS, GLONASS
Sensors	Accelerometer, gyro, proximity, compass
Network	GSM/CDMA/HSPA/EVDO /LTE

**Table 3:** Algorithm and MAE for iBeacon Calibration.

Algorithm	Bias (meter)	MAE (meter)
CF	0.027	0.024
KF	0.06–0.29	0.028
CFKF	0.026	0.021
Measurement	0.24–0.51	0.156

**Table 4:** RSSI for Different Distances.

Distance (Meters)	1	2	3	4	5	6	7	8	9	10	12	15
RSSI (dBm)	−88.9	−96.5	−99.8	−101.2	−102.4	−102.8	−103.3	−103.2	−103.5	−103.4	−104.1	−103.8

**Table 5:** Algorithm and MAE for iBeacon Field Experiment.

Algorithm	Bias (meter)	MAE (meter)
CF	0.049	0.25
KF	0.06–0.15	0.29
CFKF	0.045	0.09
Measurement	0.35–0.67	0.47

**Table 6:** Algorithm and MAE for iBeacon Field Experiment in Large Area.

Algorithm	Bias (meter)	MAE (meter)
CF	0.28	0.27
KF	0.11–0.45	0.28
CFKF	0.26	0.26
Measurement	4.72–5.71	2.36

## Data Availability

Not applicable.

## Nomenclature

**Symbol**	**Meaning**
$K_{n}$	Kalman Gain
$P_{n,n-1}$	Extrapolated Estimate Uncertainty
$R_{n}$	Measurement Uncertainty
${\hat{x}}_{1,0}$	Initial System State
$P_{1,0}$	Initial State Uncertainty
$P_{n,n}$	Estimate Uncertainty
${\;z}_{n}$	Measured System State
${\hat{x}}_{n,n}$	System State Estimate
${\hat{x}}_{n,n-1}$	Previous System State Estimate
H	Observation Matrix
β	Scale Factor
ε	Bias
